# Study of the behavior of *Euglena viridis*, *Euglena gracilis* and *Lepadella patella* cultured in all-glass microaquarium

**DOI:** 10.1007/s10544-017-0205-0

**Published:** 2017-07-07

**Authors:** Agnieszka Podwin, Wojciech Kubicki, Jan A. Dziuban

**Affiliations:** Faculty of Microsystem Electronics and Photonics, Wrocław University of Science and Technology, 11/17 Janiszewskiego St, 50-372 Wrocław, Poland

**Keywords:** Lab-on-a-chip, Glass micromachining, Cell culture, *Euglena gracilis*, *Euglena viridis*, *Lepadella patella*

## Abstract

In the paper, the microaquarium fabricated in a form of entirely glass lab-on-a-chip for culturing and microscale study of microorganisms has been presented. A new approach towards cellular studies that brings a significant improvement over commonly utilized – polymer-based solutions has been shown. For the first time, all-borosilicate glass chip was applied for the culturing of the selected microorganisms and enabled notable population growth and behaviorism investigation. The chip fabrication method in comparison to typical glass chip technology was notably simplified, including quick patterning and low temperature bonding in 80 °C. In the studies, both a single-cell (*Euglena gracilis* and *Euglena viridis)* and multi-cell microorganisms (*Lepadella patella)* were cultured in the microaquarium. Behaviorism of the selected microorganisms was investigated by supplying various proportions of carbon dioxide, nitrogen and air into the chip. Tests included studies of microorganisms chemotaxis, viability (mostly based on photosynthesis process) and coexistence in the lab-on-a-chip environment. The experiments confirmed that the developed chip is a tool that fits the requirements for the culturing and behavioral studies of microorganisms and constitute ground-works to propel its further application in broadly defined cellular study field.

## Introduction and motivation

Recently, dynamic development of lab-on-a-chip (LOC) solutions for cell culturing has been observed (Gan et al. 2011; Chawla et al. 2015). The microfluidic structure of LOC provides precise process control, minimization of reagents usage and sample protection from the contamination. These features are desired and may bring new opportunities to the cellular study fields. The commonly utilized methods of eukaryotic or prokaryotic cell culturing and characterization are being progressively preempted by such functional microfluidic devices (Hosseini et al. 2015; Christakou et al. 2015). New solutions of LOCs for cell cultures that provide well defined cell stimulation (Wang et al. 2011; Mross et al. 2015), real-time on-chip analysis of small cell cultures (Utz et al. 2015; Tsugane and Suzuki 2015), as well as a single cell management are being developed (Reinecke et al. 2015; Mogami et al. 2015; Shi et al. 2011). The literature on the subject shows comprehensive systems for cell cultures and their monitoring utilizing microfluidic-based tools. Specified chip structure may be tailored to the needs of examined cells, e.g. by creating chemical gradients within the chamber area that imitates the cell physiological environment (Ozasa et al. 2015; Ozasa et al. 2013; Forry and Locascio 2011).

Lab-on-a-chips intended for microaquatic cell culturing and investigation are often defined as “microaquaria” (Ozasa et al. 2015; Ozasa et al. 2013; Ozasa et al. 2014). The critical issue for manufacture of microaquarium is the selection of construction material, as it shall be characterized by high bio-inertness, relatively simple fabrication and opportunities to implement optical detection methods. Most of such LOCs are based on polymers, mainly polydimethylsiloxane (PDMS), due to comparatively simple micropatterning (Comina et al. 2014; Kamei et al. 2015; Hwang et al. 2015), high accessibility and moderate biocompatibility (Chang et al. 2015; Woodruff and Maerkl 2016). Another significant feature of PDMS-based solutions is the possibility to isolate the cell culture and deliver the nutrition indirectly – by the diffusion through the semi-permeable PDMS membrane (Ozasa et al. 2015). Nevertheless, some PDMS features still cannot be accepted in cellular studies (Regehr et al. 2009; Su et al. 2011; Piruska et al. 2005; Halldorsson et al. 2015; Probst et al. 2013; Lee et al. 2003; Heo et al. 2007; Mukhopadhyay 2007). One of them is affecting purity of culture buffer by leaching of uncured oligomers from the polymer network into culturing area (Regehr et al. 2009), as a result of incomplete crosslinking of improperly mixed prepolymer. PDMS also absorbs nonpolar hydrophobic molecules from the media into the polymer bulk that may strongly affect the culturing conditions by the depletion of cell culturing buffer (Su et al. 2011). Concerning commonly used in macroscale studies fluorometric cell detection methods PDMS shows significant autofluorescence in comparison with other materials (Piruska et al. 2005). Moreover, frequently applied impermanent bonding of PDMS layers with glass (Ozasa et al. 2013; Probst et al. 2013; Lee et al. 2003) inhibits high-throughput investigation, due to evaporation of water from the culture area, resulting in changing of buffer concentration and osmolality (Heo et al. 2007; Mukhopadhyay 2007). Permanent bonding of PDMS may be achieved by UV light-based bonding (Niu et al. 2006) or using thermal process after oxygen plasma activation (Plecis and Chen 2007), but these methods require more advanced technology (Haubert et al. 2006). Evaporation of liquids in PDMS LOCs has been intensively discussed in the literature recently. As a result, methods, like supersaturation of PDMS with water by soaking it in a bath of heated water (Randall and Doyle 2005) and appliance of parylene (Shin et al. 2003) or Teflon coatings (Lau and Gleason 2000) have been proposed to mitigate it. Possible diffusion of gases and various particles through PDMS membrane may have unspecified and undesirable impact on cell investigation and culture stimulation with defined factors. Therefore, despite easy-to-apply technology, PDMS microaquarium shows several drawbacks which may have negative impact on reliability of cell study in PDMS chips. Searching for the solution to this problem, we have returned to the origins of cell culturing methods that are utilized in macroscale tools and proposed in this work all-glass microaquarium.

Since 1907 culture studies have been conducted in Petri dishes that are in general mostly fabricated out of polystyrene (PS) or glass (Berthier et al. 2012). These materials are well-characterized, bio-inert and inhibit evaporation from the culture area (Berthier et al. 2012) that may provide the finest viability of cell culture, considering development of microaquarium. Nevertheless, PS or glass have not been widely utilized for on-chip cell culture so far. In the case of PS, the reason is complex and expensive microfabrication of prototypes and only few solutions have been developed recently (Nargang et al. 2014; Vasdekis et al. 2072).

All–glass microaquaria are also hardly encountered in the literature, in addition the available solutions require sophisticated technology, e.g. using rare photostructurable Foturan glass (Hanada et al. 2008; Tantawi et al. 2013). Despite glass is a main LOC construction material for other biological research, i.e. genetic or proteomic analysis, according to the RSC database, characterization and applicability of all-glass microaquarium for cell culture research has not been investigated to date. On the contrary to exclusively PDMS-based solutions, Pyrex-like borosilicate glass is entirely bio-inert and commonly applied in cell culturing tools, utensils and glass slides for microscopic observations. Its other advantages, like high endurance, chemical resistivity, unique thermal properties, and a very good optical transmission in a VIS range, make this material an interesting base for a new generation of LOCs for cell culture research. The main restriction is relatively complex standard glass microfabrication methods, especially requiring high temperature bonding (>600 °C) for combination of glass substrates. Nevertheless, our technological studies show that some of these limitations may be overcome by notable reduction of bonding temperature to 80 °C.

The comparison of PDMS and glass material features, which are significant in cell culture studies and fabrication of chip is shown in the Table 1.

**Table 1:** The comparison of PDMS and glass as construction materials for fabrication of microaquarium LOCs (Ozasa et al. 2015; Ozasa et al. 2013; Forry and Locascio 2011; Su et al. 2011; Berthier et al. 2012)

Material Feature	PDMS	Borosilicate glass
Cell culture viability	moderate/good	good
Gas permeability	good	none
VIS range optical transparency	good	best
Surface functionalization	required	none

In consequence, we have come to the basic concepts of cell culturing and in this work developed entirely glass lab-on-a-chip, fabricated using simple technological processes. To our best knowledge, presented here microaquarium concept is so far the first solution of all-glass LOC for the microaquatic cell culture research. The developed device has been successfully applied for the culturing and behavioral studies on *Euglena gracilis, Euglena viridis* and *Lepadella patella* subjected to the gas (carbon dioxide, nitrogen and air) stimulation, but may also be used in research towards other species as well.

## Materials and methods

The structure of glass microaquarium was designed to enable culturing and behaviorism investigation of the selected microorganisms, concerning their features, life-support requirements and swimming ability. Both *E. gracilis* and *E. viridis* were subjected to the chemical and optical stimulations in the developed glass LOCs. In the studies, both qualitative and quantitative analyses of euglena photosynthesis capability were conducted, depending on a kind of the species. *E. gracilis* was also subjected to the simultaneous gas (nitrogen and air) stimulation for the chemotaxis surveys. In the final experiment, *E. viridis* and *Lepadella patella* coexistence in the microaquarium chamber was studied.

### Lab-chip design and fabrication

**Fig. 1:**
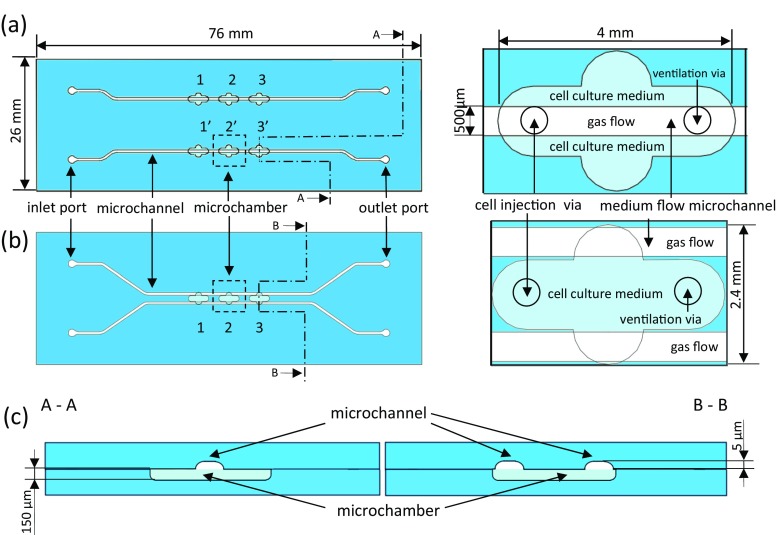
Schemes of LOCs: **a** top view of the single-side supply LOC (*left*), enlarged view of the microchamber (*right*), **b** top view of a double-side supply LOC (*left*), enlarged view of the microchamber (*right*), **c** cross-sections of microaquaria

In this paper, “microaquarium” is considered as the microfluidic system comprising cell culturing chamber(s) and microchannel(s) intended for delivery of nutrition. The appliance of microchannels provides new opportunities to conduct studies which are usually impossible to run in the classical Petri dish. The microaquarium enables behavior investigation of microorganisms influenced by one particular medium, but also to indicate the taxis of defined object towards one of the chemical stimuli. For this reason, two variants of delivery microchannels were applied (Fig. 1). In every lab-chip, the number of chambers was multiplied to enable multi-culturing, preserving the same experimental conditions. As a new insight into cell culture investigation, in reference to commonly utilized PDMS-based solutions, may be considered an assurance of direct contact between gas and culture buffer. All-glass microchambers provide entire separation of the culture from an external environment so that created within habitat is unequivocally defined and depends merely on the assumed culturing conditions.

In the first variant, the channel pattern allows one specified medium to be introduced into the microaquarium chamber (1a). In the second variant, two different media may be simultaneously and selectively supplied into the chamber, by the two channels passing through it (1b). The slanting shape at the beginning and at the ending of the microchannels was determined by the size of microfluidic connectors, applied in order to deliver media. Zones of life-supporting media are indicated by microchannels passing under the opposite sites of the microchamber. Within every microchamber area, vias intended for the injection of microorganisms and ventilation were applied. In order to conduct experiments simultaneously, the chamber pattern within every lab-chip was multiplied, arranged in series and defined by the number. The depth of microchannels was designed to 5 μm, which disables escaping of euglenas (minimal diameter: 8 μm (Pal and Choudhury 2014)) and much larger *Lepadella patella* (May et al. 1987) from the microchamber area. The depth of microchamber was designed to 150 μm to provide living space for microorganisms. In this way, the media supplied into the microchamber were unable to flush the studied objects outside the microchamber.

**Fig. 2:**
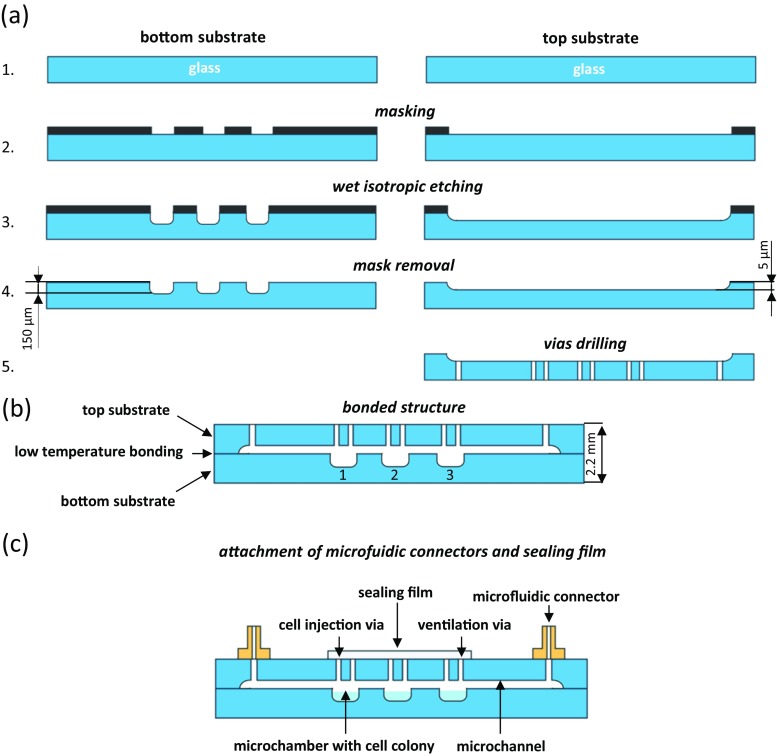
Fabrication scheme of entirely glass LOC with microaquaria: **a** preparation of the substrates, **b** bonded structure, **c** attachment of microfluidic connectors and sealing film

**Fig. 3:**
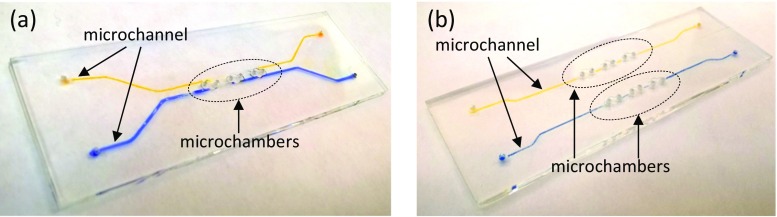
Glass LOCs with microaquaria in which microchannels are filled with orange and blue dyes to visualize the concept of selective and simultaneous delivery of various nutrition: **a** double-side supply LOC, **b** single-side supply LOC

The glass chips were manufactured utilizing processes of wet isotropic etching, mechanical drilling of vias and low temperature bonding of glass substrates (Fig. 2). The pattern of microfluidic structures was designed in CAD software and cut on an adhesive plotter foil (Avery Graph), which is resistant to HF. In our case, patterns were cut using CNC plotter with integrated 50 mW laser, however patterning may be also performed using standard cutting plotter or manually. The glass slides (Borofloat® 33, Schott) were cleaned with detergent, acetone and isopropanol (IPA), respectively. The pattern was etched through the etching window in the foil, by submerging glass slides in 50% HF:69% HNO_3_ (10:1 *v*/v) solution for required time (etching rate: ~3 μm/min). Vias for the injection of microorganisms, ventilation and gas supply were drilled in the top substrate. Prior to bonding, the glass slides were cleaned with acetone, IPA, trichloroethylene, deionized water and Piranha solution (H_2_SO_4_:H_2_O_2_, 3:1). After rinsing in DI water, substrates were aligned and temporarily joined. The final bonding was obtained by heating the substrates in 80 °C in a chamber oven (Fig. 3). The temperature of 80 °C was reasonable by the structure of lab-on-a-chip, which very shallow microchannels (5 μm) could have sunk in the typically utilized high temperature bonding. Next, standard microfluidic connectors (Nanoport, IDEX) were adhesively bonded to the top layer of the chip to provide easy connection of tubes with life-supporting media. After the injection of microorganisms, via holes were covered with sealing film (Excel Scientific) to protect the sample from the external environment - inhibit evaporation, autofluorescence and provide high optical clarity.

### Objects of the study

The microorganisms widely occurring in the freshwater habitat, i.e. belonging to the kingdom of protists and animals, were studied within the fabricated microaquaria. According to Whittaker’s classification, protists are one of five kingdoms, besides monera, animals, plants and fungi (Hagen 2012). They enclose diverse microorganisms that may be assigned to protozoa (e.g. *Paramecium*), algae (e.g. *Rhodophyta*) and mushroom-like representatives (e.g. *Physarum*). Within the subkingdom of algae, euglena is considered as the one of the most interesting creatures. The assignment of euglena to the algae is rather controversial, since its photosynthesis capability and simultaneous high mobility make this microorganism torn between protozoa and algae group indeed (Solomon et al. 2000).

Protists, particularly euglenas, have many new applications that have propelled the research interests towards these microorganisms. One of the most surprising news announced by *Euglena Corporation* was the possibility of obtaining the specified bio-oil from euglenas, having similar features to kerosene. Considering microscale applications, (Itoh et al. 2008) proposed utilization of euglenas for moving microparts in liquid buffers, using photophobic responses of this creature. Growing interest in euglena properties and applications has motivated us to use this microorganism in our research as well.

In the developed glass microaquaria two species of euglena, namely *Euglena gracilis* and *Euglena viridis* were cultured*.* These creatures normally live in the freshwater habitat. Their presence may be confirmed by the green color of water reservoir in which they currently exist. Typical size of *E. gracilis* is 35–50 μm in length and 8–20 μm in diameter. *E. viridis* is a bit larger, that is 40–65 μm in length and 14–20 μm in diameter (Pal and Choudhury 2014). The microaquaria were used to study photosynthesis-based development of euglenas, as well as the influence of gas media on *E. gracilis*, i.e. air and nitrogen, which are necessary for the microorganisms growth in any culture, as indicated in (Carlson 2010).

Additional research was conducted for the representative of animal kingdom, *Lepadella patella*. This creature is about five times bigger than euglena, with typical length up to 220 μm (May et al. 1987). On the contrary to euglena, which is an autotroph, *L. patella* is a heterotrophic microorganism and cannot use the energy from light to produce organic compounds (Glime 2013; Streit et al. 1997). In our experiments, *L. patella* (predator) and *E. viridis* (prey) were cultured in the same microchamber and stimulated by constant nitrogen and air flow. As a result of experiments, mutual relationship between the selected microorganisms was investigated.

### Culture preparation

Commercially available *E. gracilis* living specimen was applied and inoculated into the medium based on the wheat and rice, according to the guidelines (Blades Biological Ltd., Protozoa and algae culture instructions).

*E. viridis* colony was collected from its natural habitat, salty-sweet water sample from Puck Bay shore (Baltic, Poland) between March and April. The maintenance of macroscale *E. viridis* culture was supported by replenishing it with fresh tap water and adding a fresh blade of hay once a week.

*L. patella* was separated from the water sample, obtained from the Odra river (Wrocław, Poland) in April. The maintenance of *L. patella* colony in the medium was achieved by using a procedure applied for *E. viridis*.

### Measurement set-up

**Fig. 4:**
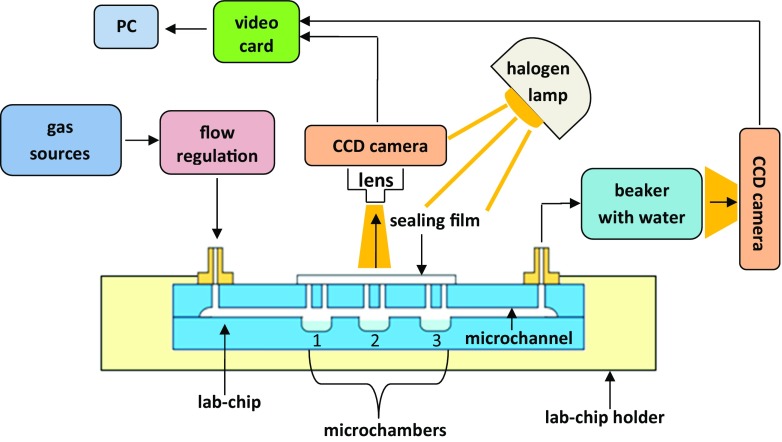
Scheme of the microorganisms study in microaquaria – measurement set-up

In the experiments, carbon dioxide (99.9%), nitrogen (99.99%) and air (99.99%) were delivered into the LOCs using tubes and pneumatic regulation. The gas sources (Air Products, Poland) were combined with pneumatic line, equipped with reducers and throttles arranged in series to maintain the regulated flow rate within the microaquaria. Before the gases were delivered to the lab-chips with cultures of microorganisms, the value of flow rate was confirmed using bubble detection method. At the output, quantity of bubbles per unit time and bubble volume was observed by the CCD camera and next recorded image was used to define the flow rate. Photosynthesis tests were carried out using white light of halogen lamp (40 W, 600 lm). The observation of microorganisms behavior and taxis confined in the glass lab-chips was carried out under the optical microscope (NJF-120A, DELTA Optical) with the integrated CCD camera and visualized on PC computer using dedicated application. Each lab-chip was placed in the 3D printed holder to obtain repeatable illumination and optimized conditions of video signal. The microorganisms were sampled from the macroscale culture and introduced into the microaquarium chamber using standard laboratory pipette. After this step, microchamber vias were covered with sealing film. The microaquarium image was acquired by the camera every 24 h. On the basis of recorded image sequence, behavior of the microorganisms was investigated. The overall scheme of the measurement set-up used in experiments is shown in the Fig. 4.

**Fig. 5:**
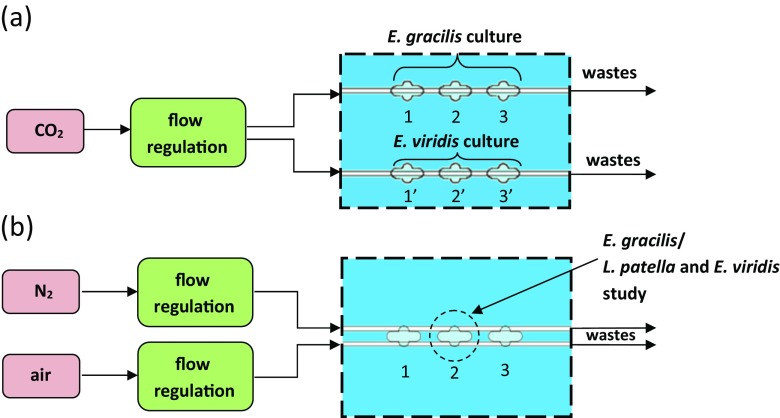
Experimental set-up schemes for microorganisms stimulation in dedicated LOCs with regard to planned experiments: **a** photosynthesis tests on euglena species viability, **b** chemotaxis experiment on *E. gracilis*/study of relationship between *L. patella* and *E. viridis*

The experiment conditions for the investigated microorganisms were established as follows. Lab-chips filled with the culture buffer were stored in ambient temperature and lightened with halogen light, merely in the case of photosynthesis tests. The first experiment concerned the euglena photosynthesis-based development. Two species of euglena were separately injected into the chip microchambers characterized by the single-side supply of the selected gas. Carbon dioxide was introduced into the microchannels with a flow rate 4 μl/s, and the halogen lamp illumination was applied to enable the photosynthesis. The subsequent tests were conducted to study *E. gracilis* chemotaxis towards gas media - air and N_2_ (flow rate: 10 μl/s), which were separately delivered into the second microchamber of the chip with double-side supply of the selected gases. The last experiment pertained to the mutual relationship between *E. viridis* and *L. patella*. Both types of the microorganisms were introduced into the microchamber (2) and affected by contemporaneous N_2_ and air supply (flow rate: 10 μl/s) to compare their survivability within the created environment. The concept of media supply and stimulation on chosen microorganisms in planned experiments is presented in the Fig. 5.

## Results

### Photosynthesis studies on *E. viridis* and *E. gracilis*

**Fig. 6:**
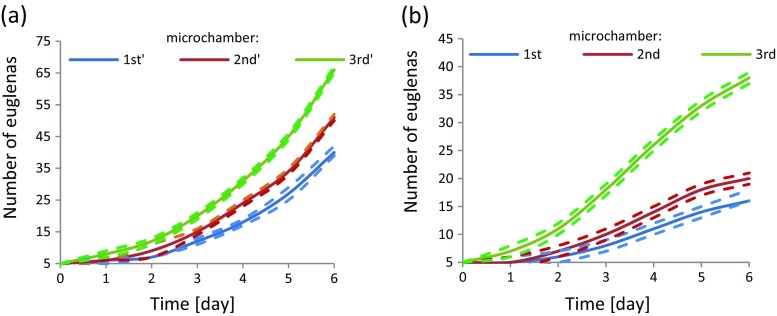
The development of euglenas in the glass LOC with regard to microchamber placement: **a** *E. viridis* population growth characteristics, **b** *E. gracilis* population growth characteristics. CO_2_ flow rate: 4 μl/s, lab-chip was constantly illuminated by halogen lamp. Data collected from 3 tests, in which *dashed lines* define maximum and minimum values respectively, while *solid lines* correspond to mean value

In the preliminary study we have investigated photosynthesis capability of euglenas. The microorganisms in the initial number of five were injected into the chip microchambers and the optical and CO_2_ stimulations were applied. The experiments were repeated three times in the same conditions. The development of euglenas population was very similar. It was noted that within six days of experiment, *Euglena viridis* colony increased in every chip microchamber, but not equally. The highest population growth was observed in the microchamber 3′ – the furthest from the gas inlet. The results of experiments on *E. viridis* colony development within the microaquarium with regard to the microchamber placement are shown in the Fig. 6a.

In the studies of *E. gracilis*, the photosynthesis-based development was also observed. After five days of culturing, its population increased from 5 to 16, 20 and 38 in consecutive chambers from gas inlet. Similarly to *E. viridis,* the greatest colony growth in this case was noted in the furthest microchamber. However, with regard to illustrated graph (Fig. 6b), it may be noticed that on the contrary to *E. viridis,* at the fourth day of experiment, *E. gracilis* culture development loses its exponential character. It indicates that this species in comparison with *E. viridis* reveals a bit different culturing conditions that may be the result of its lower chlorophyll content and consequently, limited ability to photosynthetize.

Noticed *E. gracilis* behaviorism in photosynthesis test is contradictory to the findings described in Ozasa’s works (Ozasa et al. 2013). The paper mentions that only solution of <15% CO_2_ in N_2_ may positively influence *E. gracilis*, while higher concentration of this gas results in their escape or freezing in the CO_2_ delivery area. Our experiment shows that in proposed lab-chip solution, euglenas may fission in 99.9% CO_2_ concentration as well. Results of euglena colony development in our microaquarium may be compared to the macroscale culture of this species described by M. Cramer and J. Myers (Cramer and Myers 1952), who also observed its exponential growth in the five-day experiment, concerning stimulation with carbon dioxide and light.

### Chemotaxis experiment on *E. gracilis*

**Fig. 7:**
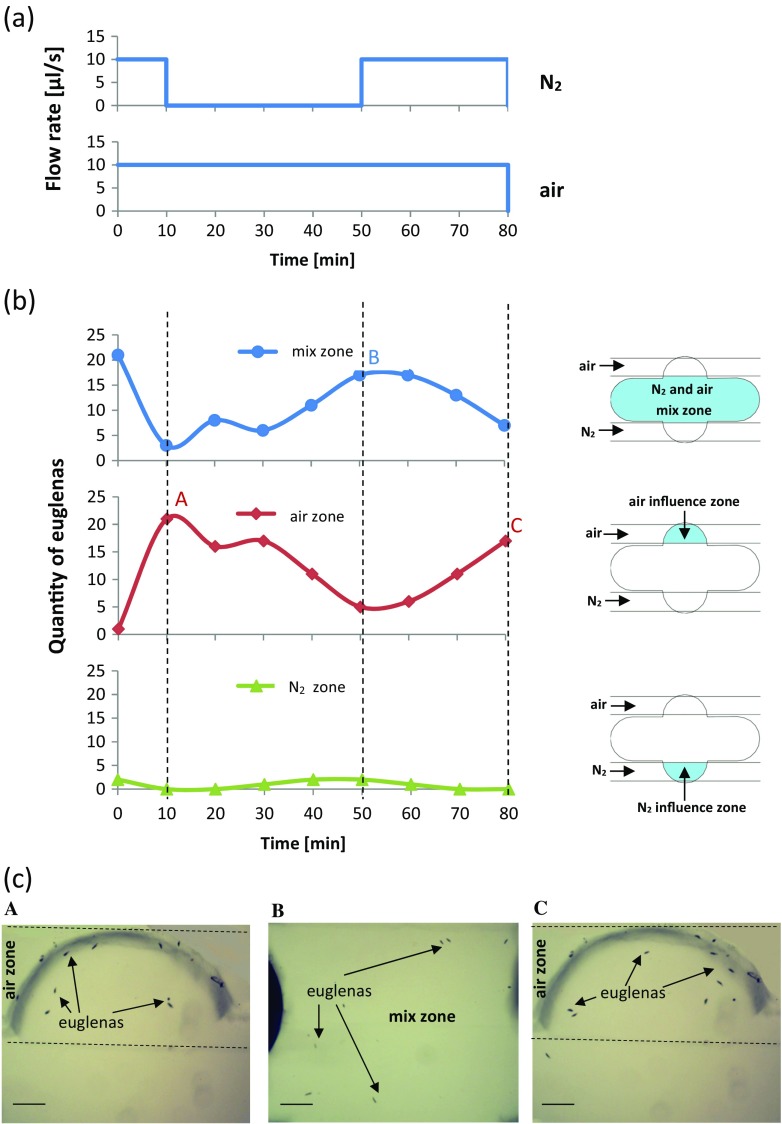
*Euglena gracilis* migration and behavior in the microaquarium chamber in response to applied gas stimulation: **a** characteristics of air and N_2_ stimulation in the microchamber, **b** response of euglenas to set gas stimulation, indicated by their observed quantity in different zones of microaquarium chamber, **c** microscopic images of euglenas distribution in the microaquarium: *a* euglenas gathered in air zone 10 min after exposure to air and N_2_, *b* euglenas gathered in the mix zone in 50th minute of experiment, *c* euglenas gathered in air zone in 80th minute of experiment. Lab-chip was stored in ambient temperature, lightened by daylight. Scale bar – 100 μm

Chemotaxis experiment on *E. gracilis* was conducted to investigate the reaction of this microorganism to the chemical stimuli. In this test, twenty four euglenas were injected into the microchamber 2 with double-side supply of the selected gases. The creatures were randomly distributed within the microchamber area. A minute later, one of the microchannels was filled with pure air, while another with pure nitrogen. Flow rates were set to equal. Seven minutes after the introduction of gas stimulation, colony shifted to the air delivery area. The behavior of investigated microorganisms was recurrent. After disconnection of N_2_, the euglenas started to migrate and swim through the other parts of the microchamber as well, but when N_2_ was applied again, the positive chemotaxis towards air returned. The characterization of migration behavior of euglenas within the developed microaquarium is presented in details in the Fig. 7.

### *E. viridis* and *L. patella* coexistence and survivability

**Fig. 8:**
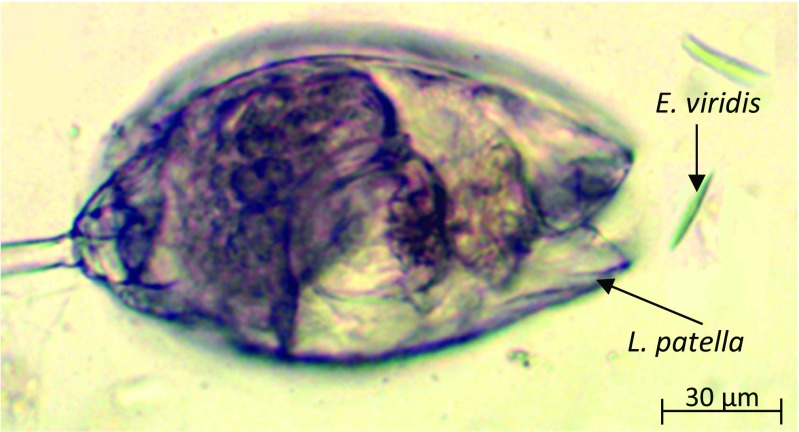
*L. patella* feeding on *E. viridis* within the microaquarium chamber

Another experiment was conducted to investigate the mutual relationship between *Euglena viridis* and *Lepadella patella,* triggered constantly by N_2_ and air flow. The microorganisms were injected into the microchamber in the initial value of 15 (*E. viridis*) and 5 (*L. patella*). Afterwards, equal gas stimulation was applied. At the beginning, the test showed the domination of *L. patella* towards euglena. *E. viridis* were consumed by *L. patella*, slowing down the development of euglena colony (Fig. 8). Nevertheless, further investigation showed that after three days of experiment, *L. patella* died out in the created environment, while *E. viridis* colony still existed and the population of this microorganism increased from 15 to 44. The characterization of the microorganisms colony development is presented in the Table 2.

**Table 2:** The characterization of *E. viridis* and *L. patella* colony development within the microaquarium chamber (experiment conditions: N_2_ and air flow rates: 10 μl/s, lab-chip was stored in ambient temperature and lightened by daylight)

Time [hours]	Number of microorganisms		Notes
	*Euglena viridis*	*Lepadella patella*	
0	15	5	Start of the test
24	17	5	Lepadellas feed on euglenas and slow down their colony development
48	25	3	Lepadellas population start to decrease – euglenas colony is able to grow faster
72	35	0	Lepadellas die out – euglenas population notably increases
96	44	0	End of the test

## Discussion

Presented here all-glass microaquaria were found as useful tools for the study of the selected microorganisms. We have successfully cultured *Euglena viridis* that was fissioned influenced by CO_2_ and light illumination. Within six days of experiment, its population grew on average from 5 to 40 in the case of the first microchamber, from 5 to 51 for the second, and from 5 to 66 for the last microchamber. Referring to *E. gracilis* photosynthesis capability, the colony growth of this microorganism was also observed but was not as high as for *E. viridis*. At the fourth day of culturing, *E. gracilis* multiplication activity decreased significantly, that may be the result of inadequate culturing conditions that refer to the specificity of this species (different amount of chlorophyll, limited photosynthesis capability). For both species of euglena, the best colony development was noted in the microchamber placed the furthest to the gas inlet. It may be caused by microfluidic impedance within the chip, which holds the carbon dioxide in the microchambers situated nearer the gas inlet and provides last microchamber with better absorbed CO_2_ concentration. Mentioned phenomenon may be also connected with creation of carbonic acidic – H_2_CO_3_, which concentration in water is the highest in the microchamber 1 and due to its use up with each succesive microchamber, the best conditions for the microorganisms growth are provided in the microchamber 3. Other tests for the purpose of explanation of this phenomenon should be conducted, comprising the measurements of pH to define the possible H_2_CO_3_ concentration in the lab-on-a-chip environment. The chemotaxis experiment towards one of the gas stimuli showed *E. gracilis* repeatable “sympathy” towards air, instead of nitrogen. Switching test proved that glass chip provides proper conditions for selective delivery of chosen gases and may be successfully applied for the chemotaxis tests of other creatures as well. Within the microaquarium, authors also noted the heterotrophy of *L. patella* towards *E. viridis*. This experiment additionally showed that *E. viridis* may develop in mixed air and nitrogen habitat with flow rate equal to 10 μl/s, which is not possible for *L. patella*. Additional experiments to further investigate the biological potential of *L. patella* in glass chips are currently in progress.

## Conclusion

We have demonstrated the entirely glass microfluidic device as a new tool towards studies of microorganisms, namely *Euglena viridis*, *Euglena gracilis* and *Lepadella patella* in a microscale. The proposed microaquaria provided adequate conditions to establish the cultures and study their behaviorism and taxis in response to various gas media, including carbon dioxide, nitrogen and air. Glass chip structure avoided evaporation and guaranteed the long-term water maintenance within the microchamber, in which no water supply was introduced. It is essential for the creatures occurring in an aquatic environment and constitutes an important improvement over PDMS solutions, in which culture maintenance last even less than 1 h, if constant supply of liquid is not provided (Heo 2009). The chips enabled to selectively deliver the specified media into the culture and allowed for changeable culturing conditions of chosen creatures. The observation of microorganisms confined in the glass lab-on-a-chips covered by specified sealing foil was favorable, providing high image resolution with none autofluorescence. Proposed glass chips can be easily cleaned and reused in the experiments.

Basing on the conducted research, *E. gracilis* in comparison with *E. viridis* reveals a bit different culturing conditions, however the culture establishment, maintenance and development was possible for both species. Similarly as in the case of macroscale culture of euglena, the colony growth in our lab-chip increased exponentially. As interesting may be considered the repeatable chemotaxis of *E. gracilis* towards air, instead of (as it may seem) neutral nitrogen. The mutual coexistence of *E. viridis* and *L. patella* confined in the microaquarium chamber that proved to be regular, consistent with the literature data has also been investigated.

It may be contended that the fabrication and appliance of entirely glass lab-chip for the culturing and behavioral studies of microorganisms is advantageous and bring reliable investigation. We have presented a novel, simplified manufacture method of glass chips developed by low temperature (80 °C) bonding that may be easily and successfully exploited for the studies of various biological objects. The use of glass chip may constitute the relevant compromise between engineers, for whom fabrication simplicity is important, and microbiologists, who expect dependable cell study. Presented here preliminary results are promising and further research towards cells’ viability and behaviorism in other culturing conditions are under scrutinizing in our laboratory.
